# Distributed multi-camera multi-target association for real-time tracking

**DOI:** 10.1038/s41598-022-15000-4

**Published:** 2022-06-30

**Authors:** Senquan Yang, Fan Ding, Pu Li, Songxi Hu

**Affiliations:** 1 School of Intelligent Engineering, Shaoguan University, Shaoguan, 512005 China; 2 Foshan Nanhai Guangdong Technology University CNC Equipment Cooperative Innovation Institute, Foshan, 528225 China

**Keywords:** Electrical and electronic engineering, Software

## Abstract

Tracking and associating different views of the same target across moving cameras is challenging as its appearance, pose and scale may vary greatly. Moreover, with multiple targets a management module is needed for new targets entering and old targets exiting the field of view of each camera. To address these challenges, we propose DMMA, a Distributed Multi-camera Multi-target Association for real-time tracking that employs a target management module coupled with a local data-structure containing the information on the targets. The target management module shares appearance and label information for each known target for inter-camera association. DMMA is designed as a distributed target association that allows a camera to join at any time, does not require cross-camera calibration, and can deal with target appearance and disappearance. The various parts of DMMA are validated using benchmark datasets and evaluation criteria. Moreover, we introduce a new mobile-camera dataset comprising six different scenes with moving cameras and objects, where DMMA achieves 92% MCTA on average. Experimental results show that the proposed tracker achieves a good association accuracy and speed trade-off by working at 32 frames per second (fps) with high definition (HD) videos.

## Introduction

The availability of new technologies such as remotely-operated and autonomous drones, wearable visual sensing equipment, and ground robots, allow a rapid deployment of mobile cameras in unknown environments with the ability to adapt to unforeseen situations, extend the duration of an observation and improve the performance of video analysis^1^. Moreover, the increasing need for safety and security, combined with the growing availability of these visual sensors mounted on mobile agents, make camera networks increasingly explored^2^. Applications include public and private environments, such as robot navigation in post-disaster areas, crime prevention, traffic control, autonomous driving, accident detection, and monitoring patients, elderly, and children at home^3,4^.

In order to automatize the interaction between humans and the surrounding environment, mobile cameras require to find the objects of interest (*detection*), follow them by an over-time localization (*intra-camera tracking*), and link the same objects across the camera network (*re-identification*) by exploiting the redundancy and richness of information provided by all cameras. We define this overall task as object *association* which is normally performed by employing each single camera with the aim of monitoring an area as wide as possible.

**Figure 1:**
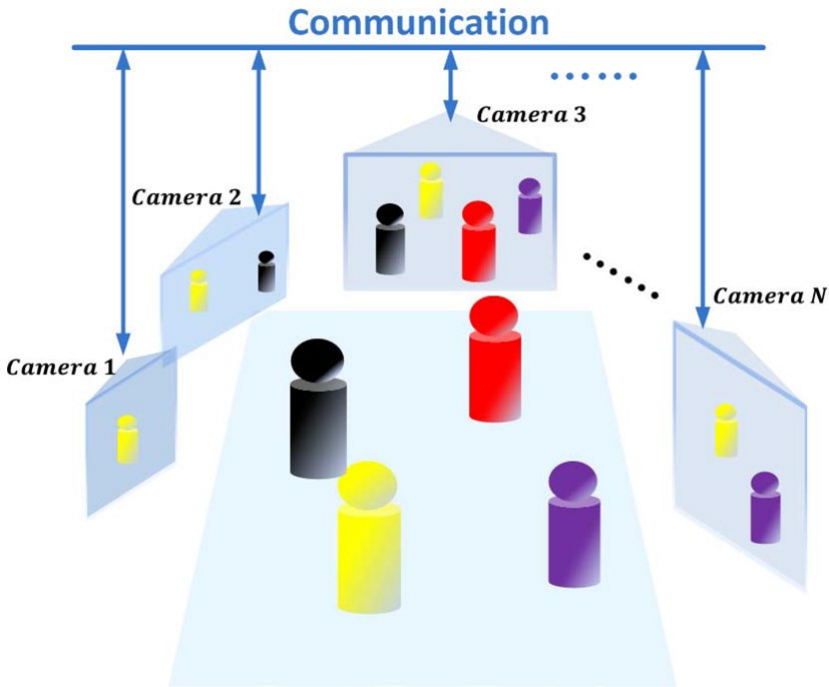
Pictorial layout of a camera network. Each camera unit is a node in the network. Cameras can see different people at a certain time instant. Blue lines correspond to communication links.

When association in a camera network is performed with cameras presenting both overlapping and non-overlapping Fields-of-Views (FoVs), the task-at-hand has to face constant changes in illumination and background both locally and across cameras without the possibility of reliably calibrating the cameras for position (viewpoint) and color. Targets can then appear and be seen from different viewing angles, thus making challenging association and assignment of unique IDs that are robust to frequent entering and exiting of the cameras’ FOVs. In addition to this, time efficiency is fundamental when deploying mobile cameras due to the nature of the dynamic interactions between humans and environment^5^. This can be achieved by having both an efficient communication across the network robust to mis-communications^2^, and a fast on-board implementation of the association algorithm. For example, in forensic applications decisions must be taken immediately when an event occurs and suspects have to be followed continuously over time. A camera network is also required to be resilient to different network sizes and must be able to integrate new cameras joining the network, with a fully distributed approach being favourable to avoid single failure points^6^. Figure 1 shows a typical mobile camera scenario.

**Figure 2:**
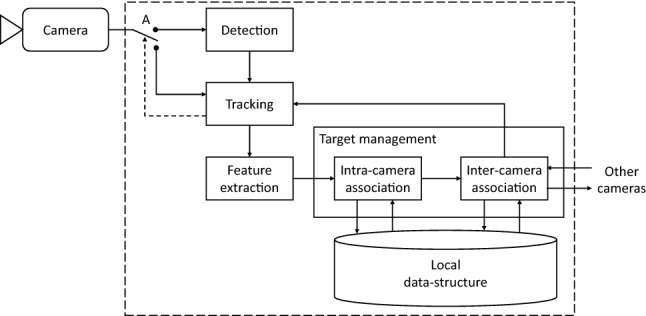
Block diagram of the Distributed Multi-camera Multi-target Association (DMMA). A: switch activated periodically or when the tracking confidence is low. Target management: receives in input the extracted features; deals with intra-camera and inter-camera associations, both by the Hungarian algorithm^7^; communicates with the other cameras. Local data-structure is updated at each time step.

In this paper, we propose DMMA, a real-time target-management module for Distributed Multi-camera Multi-target Association, a distributed strategy suitable for moving cameras (see Fig. 2). The management module updates and shares across the network a data-structure that maintains target labels and appearance over time using local and network information to obtain robustness to both occlusions and target appearance/disappearance. Moreover, a new camera joining the network can be fully operational after downloading the data-structure from the other nodes. A consensus among the cameras is obtained by sharing the data-structure variations across the network with decisions taken locally during association.

In summary, our main contributions are:

- a target-representation that consists of both appearance and deep features;
- a target-management module that deals with occlusions *as well as* targets entering/exiting the camera’s FOVs;
- a novel mobile-camera dataset comprising six different scenes with moving cameras and objects.

## Related work

Target association in cameras networks deals with detection^8^, tracking^9^, re-identification^10^ and distributed protocols^11^. We provide an overview of the main methods with a focus on those solutions designed for real-time implementations.

**Camera networks** Strategies for target association in camera networks can be categorized into centralized, distributed, and decentralized^11^. Most camera networks utilize a centralized approach where a server receives data from each camera in the network^12^. Although this strategy can exploit directly existing single-camera protocols (e.g. a single-camera tracker) by fusing the information centrally, the presence of a single fusion center leads to a lack of scalability and possibly to a communication bottleneck^13^. Distributed approaches operate with no fusion centers, thus improving the scalability and potentially reducing the communication bottlenecks. However, they are normally more complex protocols as they require to reach a consensus remotely. Distributed approaches for camera networks include a multi-target square-root cubature information consensus filter to increase tracking accuracy and stability^14^ and an information weighted consensus filter for solving the data association problem^15^. Decentralized protocols instead are a hybrid solution between centralized and distributed, as cameras are grouped into clusters and they communicate with their local fusion centers only^16^. This solution may provide a more scalable solution than a fully centralized approach but less than a distributed. Schwager et al.^17^ present a strategy for the deployment of robotic cameras in a decentralized way, which can accommodate groups of cameras to monitor an environment. The majority of the solutions for camera networks focus on improving communication and how information are managed across the camera network while assuming targets are perfectly detected, tracked and re-identified^18,12,19^. However this may not be always the case. Graph modeling is an effective way to tackle object re-identification when the topology of camera network is known. Chen et al.^12^ introduced a global graph model with in input different observations, such as detections, tracklets, trajectories or pairs. Cai et al.^18^ utilized the topology information of a camera network to re-identify objects across camera views. Hofmann et al.^19^ presented a global min-cost flow graph that joins the different-view detections.

**Detection** In order to properly associate multiple targets across a camera network, targets require to be detected in each of the cameras where they are visible^20^. Mobile cameras are challenging for background subtraction techniques since the background constantly changes, hence approaches based on learning the shape of the target are normally preferable^21^. Single-Shot Detector (SSD)^22^, You Only Look Once (YOLO)^23^ MobileNet^24^ and EfficientDet^25^ are examples of target detectors with implementations that can run in real time and are based on detecting a shape learned during training.

**Tracking** Once the targets are detected, an identifier (ID) is assigned to each target and ideally kept over time and across all cameras. If a target is new to the network, then a new ID is created. Tracking and re-identification deal with assigning an ID in a single camera and across cameras, respectively, and while the main challenge of a tracker is to maintain the same ID to the same target over time, re-identification focuses on assigning the same ID to the same target seen by different cameras. A Multi-Object Tracking (MOT) framework for mobile cameras was proposed by Choi et al.^26^ where both the camera’s ego-motion and the objects’ paths are estimated. Detections can be linked with Markov Decision Processes (MDP)^27^, a Kalman filtering in the image space along with a frame-by-frame data association based on the Hungarian algorithm and weights obtained by the amount of bounding-box overlap (SORT)^28^, or by a Convolutional Neural Network (CNN)^29^. Graph-learning based methods^30,31^ are effective in associating trajectories for the targets, but tend to fail in occlusion scenario. This problem can be dealt with by learning and updating the appearance of targets using a track management^32^ or a person re-identification dataset^33^. In order to increase robustness, a self-supervised learning detector can be employed by combining re-identification feature^34^ or by using the prediction of the motion^35^.

**Re-identification** Re-identification techniques deal with illumination changes, and variations of viewpoint and pose, by extracting robust visual features describing the target, including color^36^, texture^37^ and shape^38^ features, or by deep learning^39^. The latter methods are normally more effective as they are capable of obtaining the most discriminative features for the targets, although they fail in scenarios different from the training set. A solution to this is reinforcement learning which allows an algorithm trained on a dataset to be tested on another dataset^40^. An unsupervised cross-dataset transfer learning approach was proposed in^41^, where an asymmetric multi-task dictionary model was learned to extract discriminative features from an unlabelled target data. Cheng et al.^42^ introduced a transfer-metric learning approach with a shared latent subspace to describe the commonalities of persons in different datasets. Wang et al.^43^ proposed a transferable joint attribute-identity deep learning, which simultaneously learns attributed labels and identity features across different datasets.

Compared to the state-of-the-art methods, we deal with association by relying on a local database shared across the network in order to deal with continues changes of the appearance of a target and with cameras entering/exiting the network. Moreover, our algorithmic choices are made to optimize speed and enable a real-time implementation.

## Proposed approach

### Overview

Let ${\mathcal {C}} = \left\{ C_1,\ldots ,C_c, \ldots ,C_N\right\}$ be a network with *N* cameras and ${\mathcal {L}}=\{l_1, \ldots , l_l, \ldots l_L\}$ be the set of possible target labels. Each camera $C_c$ has a local data-structure that stores the features for each target for the past *J* frames and is maintained up-to-date over time.

In order to operate in real time, a target-management module in each camera optimizes the assignment of the labels to the targets over time, and manages cameras leaving/joining the network.

For intra-camera tracking, each camera is equipped with target detection and tracking modules. As the latter has to be scale-invariant to cope with moving cameras and fast to maintain real-time, a trade-off has to be sought between fast trackers that may not be scale invariant^44^ and scale-invariant trackers that may be slow^45^. The target-management module performs association between existing targets and detections in each camera, and inter-camera association with the features of the targets received from other cameras.

#### Remark 1

Our focus is to implement an efficient target association while assuming an ideal communication across cameras, namely the data transmission has no loss or delay. In our experiments, cameras exchange targets information, which are wrapped by .xml files, through the computer memory. See^46^ for more details on non-ideal communication.

### Target descriptor

Let $\mathbf{x }_c^{l}(t)$ represent the features of target $l_l$ at time *t* in camera $C_c$ obtained by target detection and let a local data-structure in each $C_c$ maintains over time the features of each target for the past *J* frames. The features for target $l_l$ are defined as

1 $$\begin{aligned} \mathbf{x }_c^{l}(t)=[H_{\mathbf{x }_c^{l}(t)}, D_{\mathbf{x }_c^{l}(t)}], \end{aligned}$$

where $H_{\mathbf{x }_c^{l}(t)}$ and $D_{\mathbf{x }_c^{l}(t)}$ are the appearance and deep features of the target, respectively. $H_{\mathbf{x }_c^{l}(t)}$ concatenates two RGB *m*-bin histograms $H^1_{\mathbf{x }_c^{l}(t)}$ and $H^2_{\mathbf{x }_c^{l}(t)}$, which are obtained on image patches of upper and lower parts of a target. The bins of the histogram are defined through a computationally efficient colour-naming (*CN*) approach following the insights of^47^ that defines how CN is a strong visual attribute robust to intensity variations^48,49^ when the discriminative RGB values are learned directly from public datasets.

Similarly to^47^, we choose *m* = 11 for its discriminating accuracy with bins representing black, blue, brown, grey, green, orange, pink, purple, red, white and yellow colours. Unlike^50^ that employs same-size patches, we calculate the histograms on image patches with size adaptive to the target bounding box in order to deal with changes in target size. Let *M* and *N* be the bounding-box height and width, respectively, the side of an image patch is

2 $$\begin{aligned} a = \frac{\max \left\{ M,N\right\} }{2K} \end{aligned}$$

pixels. $H^1_{\mathbf{x }_c^{l}(t)}$ and $H^2_{\mathbf{x }_c^{l}(t)}$ are each obtained on *K*/2 squared image patches, whose centre ${\varvec{r}}$ is located as^50^:

3 $$\begin{aligned} {\mathcal {N}}({\varvec{r}}|\varvec{\mu } ,\varvec{\varSigma })=(2\pi )^{-\frac{K}{2}}|\varvec{\varSigma }|^{-\frac{K}{2}}e^{-\frac{K}{2}({\varvec{r}}-\varvec{\mu })^T\varvec{\varSigma }^{-1}({\varvec{r}}-\varvec{\mu })}, \end{aligned}$$

where ${\mathcal {N}}$ is a normal probability density function with mean $\varvec{\mu }=[M/2, N/2]$ and covariance matrix

4 $$\begin{aligned} \varvec{\varSigma } =\begin{bmatrix}2N&{}0\\ 0&{}3M\end{bmatrix}. \end{aligned}$$

Colour histogram feature is insensitive to pose and shape deformation variation, because it utilizes the statistical information of the target. However, as the detected target images usually include background and occlusion, the statistical feature is not robust for real-world application. Deep learning based methods have been successfully applied in extracting discriminative feature for re-identification^51^. Although these methods achieve better accuracy, they are usually time-consuming. To achieve real-time processing, we use an efficient pre-trained backbone network to extract feature. The choice of backbone is explained in detail in “Experimental results” section.

**Figure 3:**
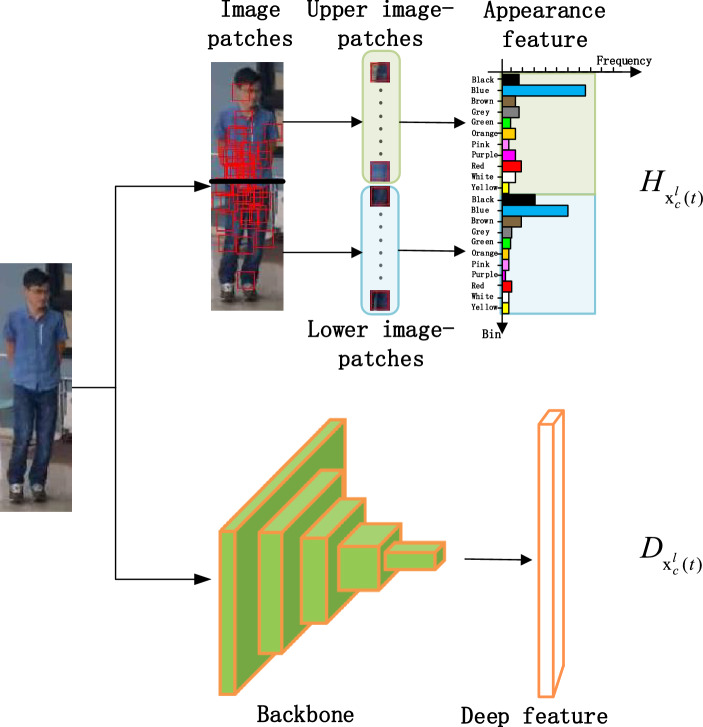
Appearance feature (top) as the concatenation of upper (light green) and lower (light blue) histograms and deep feature (bottom) extracted from a backbone network.

As shown in Fig. 3, the appearance feature $H_{\mathbf{x }_c^{l}(t)}$ concatenates upper and lower CN histograms and the deep feature $D_{\mathbf{x }_c^{l}(t)}$ is extracted from a backbone network.

### Target management

The target-management module performs association between existing targets and new target detections (intra-camera association), and between existing targets and new targets from the network (inter-camera association). The pairs of targets, *i* and *j*, considered for association are those with a high appearance-correlation

5 $$\begin{aligned} \kappa (\mathbf{x }_c^{i}(t), \mathbf{x }_c^{j}(t))>\psi , \end{aligned}$$

where $\kappa$ is the correlation function and, only for intra-camera association, spatial intersection-over-union of bounding boxes greater than $\gamma$. The more abrupt the illumination changes are expected in the scene, the lower $\psi$, and the faster the targets are expected to be and the lower the fps of the video stream is, the lower $\gamma$. Association is performed by the Hungarian Algorithm^7^ and, in intra-camera association, detections not associated are considered new targets. A consensus among cameras is obtained by performing the intra-camera association, followed by the inter-camera association. This maintains the labels consistent over time for targets meeting the appearance-correlation constraint (Eq. 5). The target management module processes sequentially the inputs received by the network and shares in the network modifications on appearance (and label).

Object features are updated in the data-structure as

6 $$\begin{aligned} \mathbf{x }_c^{l}(t+1) = (1-\alpha _f)\hat{\mathbf{x }}_c({\hat{t}}) + \alpha _f \mathbf{x }_c^{l}(t), \end{aligned}$$

for intra-camera association, where $\hat{\mathbf{x }}_c({\hat{t}})$ is the appearance feature of the associated detection, ${\hat{t}}\in \{t-J, \ldots ,t-1,t\}$ and $\alpha _f$ is the *forgetting* factor of each camera. A lower $\alpha _f$ would result in a less discriminative feature vector, while a higher $\alpha _f$ would make the tracking less responsive to appearance changes, thus producing drift.

For inter-camera association, appearance features are updated with the data received from other cameras as:

7 $$\begin{aligned} \mathbf{x }_c^{{\overline{l}}}(t+1) = (1-\alpha _n)\overline{\mathbf{x }}_{{\overline{c}}}^{{\overline{l}}}({\overline{t}}) + \alpha _n \mathbf{x }_c^{l}(t), \end{aligned}$$

where $\overline{\mathbf{x }}_{{\overline{c}}}^{{\overline{l}}}({\overline{t}})$ is the appearance feature of the associated target with label $l_{{\overline{l}}}$ from camera $C_{{\overline{c}}}$, ${\overline{t}}\in \{t-J,\ldots ,t-1,t\}$ and $\alpha _n$ is the *network* factor. The lower $\alpha _n$, the more the information from the network is considered.

## Validation

### Datasets and experimental setup

**Figure 4:**
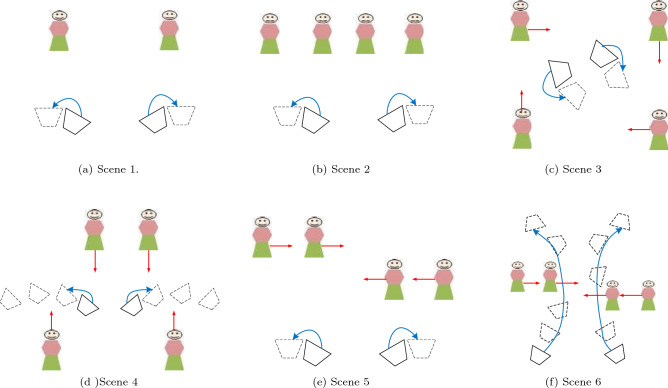
Diagrammatic overview of the proposed dataset. Legend: Trapezoid = camera; blue arrow = camera movement; red arrows = target movement.

To validate the proposed method, we decided to run our experiments on people as target. Existing camera network datasets only contain static cameras where also the cameras topology is available, like PETS2009^52^, NLPR_MCT^12^, DukeMTMC^53^, however in order to properly test the proposed method, we require a dataset with targets moving continuously across cameras. To this aim, we introduce a new dataset that contains six scenes with up to four people recorded with two moving hand-held cameras, where people are annotated with a bounding box (using vbb^54^). The diagrammatic overview of the six scenes is shown in Fig. 4. Videos are in HD (1280 $\times$ 720 pixels), running at 30 Hz and having more than 10,000 frames in total.

In Scene 1 and 2, we have static people but they continuously enter/exit the cameras’ FOVs due to the cameras motion, in Scene 3 and 4 people move and the illumination conditions change drastically, and in Scene 5 and 6 people move and occlude each other beside entering/exiting the cameras FOVs. The dataset is fully labeled. Each person in the sequences is manually annotated using the video bounding box (vbb)^54^. The annotations consist of position and size of the objects labeled with a unique ID.

For intra-camera tracking, we detect people with EfficientDet^25^ which is faster than YOLO^23^ and SSD^22^, and track them with Fast Compressive Tracking (FCT)^55^, chosen because of its speed (150 fps) and scale-invariant properties. FCT differentiates between target and background by calculating the likelihood of a nearby patch belonging to a target with an online Naive Bayes classifier. A convolution with Haar Filters^56^ generates a high-dimensional multi-scale feature vector, which is reduced by Compressive Sensing^55^. We initialize one FCT per EfficientDet detection and improve its performance by combining it with new detections obtained every $\delta$ frames or when the FCT tracking confidence, $\phi$, is lower than a threshold $\beta$. DMMA can run live but the validation in this section is performed on video datasets to allow a proper analysis. DMMA is instantiated with $\delta = 5$ frames, *J* = 2 frames, $\alpha _f=0.5$, $\alpha _n=0.2$, $\gamma =0.2$, $\psi =0.4$ and *K* = 48, and FCT with $\beta = 0.4$.

We implement all experiments using the same system, whose configuration is shown in Table 1.

**Table 1:** Configuration of experimental environment.

Item	Version
CPU	Intel Core(TM) i9-10900K 3 GHz
GPU	NVIDIA RTX 2080 SUPER 8 GB
RAM	USCORSAIR DDR4 32 GB
Operating system	Microsoft Windows10
Python	3.8
Pytorch	1.3.1
CUDA	10.2

### Performance measures

To evaluate the performance of target descriptors, we use Cumulative Matching Characteristic (CMC) curves^57^ as the evaluation criteria, which is defined as a function of Rank-*r*:

8 $$\begin{aligned} q(r)=\frac{|C(r)|}{|{\mathcal {P}}_g|}, \end{aligned}$$

where $|{\mathcal {P}}_g|$ represents the total number of images in the gallery, and the query set *C*(*r*) is defined as:

9 $$\begin{aligned} C(r)=\left\{ p_i: rank(p_i) \le r \right\} \quad \forall p_i \in {\mathcal {P}}_g. \end{aligned}$$

Since most intra-camera tracking algorithms usually use the multi-object tracking metrics as their evaluation criteria, we utilize the evaluation metrics defined in^58^. These include number of False Positives (FP), number of False Negatives (FN), number of ID Switches (IDS), number of Mostly Lost (ML) trajectories, number of Mostly Tracked (MT) trajectories, Multiple Object Tracking Accuracy (MOTA, summary of overall tracking accuracy in terms of FP, FN and IDS), and IDF1^53^, while inter-camera association with Multi-Camera object Tracking Accuracy (MCTA)^12^:

10 $$\begin{aligned} MCTA=\left( \frac{2pr}{p+r}\right) \left( 1-\frac{ \sum _{t}m_t^s}{\sum _{t}u_t^s}\right) \left( 1-\frac{ \sum _{t}m_t^c}{\sum _{t}u_t^c}\right) \end{aligned}$$

where $p = 1-\frac{\sum _{t}f_t}{\sum _{t}h_t}$ is the precision, $r = 1-\frac{\sum _{t}i_t}{\sum _{t}g_t}$ is the recall, and $m_t$, $u_t$, $f_t$, $h_t$, $i_t$ and $g_t$ are the number of ID switches, true positives, false positives, trajectory hypothesises, misses and ground truths at time *t*, respectively, and where *s* and *c* denote matches within the same and across cameras, respectively. MCTA ranges between 0 and 1 (the higher MCTA, the better the performance). Speed is measured in frames per seconds (fps) on the algorithms.

### Experimental results

In this section, we firstly evaluate the target representation, the intra-camera, and the inter-camera tracking performances. Then we analyze the impact of parameters and compare with state-of-the-art methods on MOT16 dataset. Finally, the qualitative results are depicted.

**Target representation performance** Table 2 compares the appearance representation, *CN*, with the results by the Hue (*H*) and Saturation (*S*) histograms of the randomly-sampled patches projected on 30 *H* bins and 32 *S* bins concatenated (*HS*); a deep feature with accurate backbone (NASNet^59^); a effecient backbone (MobileNet^24^); and by concatenating *CN* and MobileNet (*CN* + MobileNet). Results are reported as the percentage of correctly matched pairs within a specific rank^57^ and speed, on 600 pairs of images distributed among different targets and case difficulty (e.g. due to occlusions or lighting changes) of the proposed dataset. As can be observed, the NASNet has the best performance with 94.2% of queries resulting in rank 1 correct match. *CN* + MobileNet is second with approximately 92.1% of the queries resulting in rank 1 correct match and 98.3% in the 30 top ranked. However, the speed of NASNet (12.5 fps) is two times slower than ours (28.1 fps). Thus, the proposed CN + MobileNet shows the best trade-off in terms of performance and speed.

**Table 2:** Comparison of appearance and deep features (see “Experimental results” section for details).

		*HS*	*CN*	NASNet^59^	MobileNet^24^	*CN* + MobileNet
%	Rank-1	59.3	66.1	94.2	91.1	92.1
	Rank-10	81.7	84.2	97.5	96.7	97.4
	Rank-20	89.3	88.3	98.4	97.6	98.1
	Rank-30	93.0	97.5	98.7	98.1	98.3
Speed (fps)		58.6	49.4	12.5	36.4	28.1

Correctly matched pairs over 600 pairs in a specific rank and execution speed. *CN*: Colour Naming; *H* Hue; *S* Saturation. *CN* + MobileNet has the best performance trade-off.

**Figure 5:**
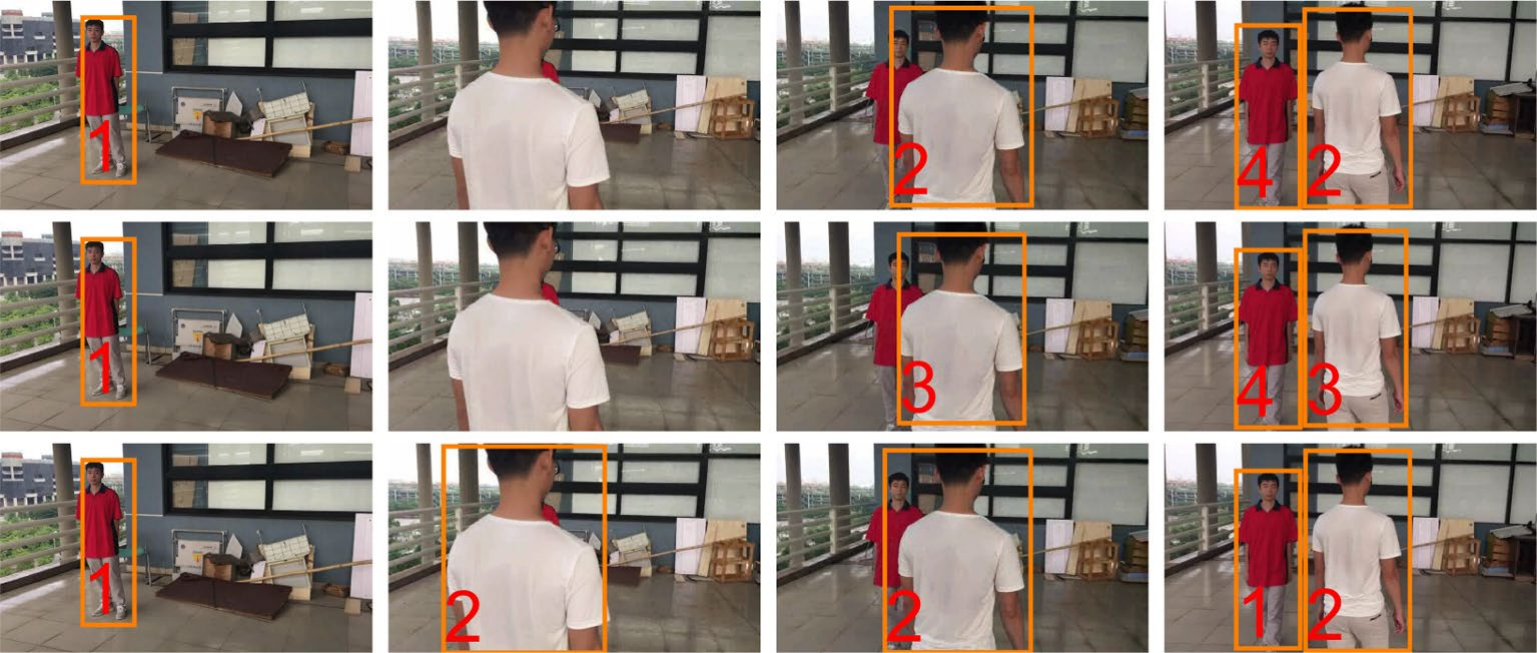
Intra-camera tracking comparison (proposed dataset: scene 2 and camera 2) with target-size changes and one heavy occlusion. Top to bottom: DeepSORT^29^, MDP^27^ and DMMA-nc. Left to right: frames 1, 190, 203 and 220. DeepSORT and MDP wrongly assign labels 3–4.

**Intra-camera tracking performance** We compare the proposed method against DeepSORT^29^, MDP^27^, MFI_tst^35^ and FairMOT^34^, for intra-camera tracking. As DMMA would use information across cameras, we perform a comparison with DMMA run as an intra-camera tracker, such as with no inter-camera communications (DMMA-nc). We also compare DMMA against detector and Hungarian Algorithm at every frame with no FCT tracking (DMMA-nt). DMMA-nc and DMMA-nt are baselines optimized for the task-at-hand. Table 3 compares intra-camera tracking results. DMMA-nc is the only method running in real-time (32 fps), while maintaining the best average MOTA. In the most difficult scenes in terms of colour changes and heavy occlusions (scenes 3, 5 and 6), DeepSORT drops accuracy with respect to MDP and DMMA-nc, while FairMOT shows comparable results with respect to DMMA-nc but cannot reach a real-time performance. Where FairMOT and MDP have a higher MOTA, DMMA-nc has a comparable accuracy. Figure 5 shows sample tracking results on the proposed datasets.

**Table 3:** Comparison of intra-camera tracking accuracy on the proposed dataset, and speed of detection and tracking combined.

		DeepSORT^29^	MDP^27^	MFI_tst^35^	FairMOT^34^	DMMA-nt	DMMA-nc
MOTA	S1	95.5	91.9	93.2	**95.7**	94.9	94.5
	S2	97.8	95.7	97.0	**98.1**	96.2	97.1
	S3	78.8	86.9	85.4	86.7	84.0	**89.3**
	S4	94.6	**96.9**	95.3	94.3	95.4	95.8
	S5	79.9	**85.8**	82.1	81.8	81.2	84.8
	S6	80.8	87.1	82.1	**89.3**	83.0	87.9
	Ave	87.9	90.7	90.3	91.1	89.1	**91.6**
	(std)	(8.1)	(4.4)	(5.3)	(5.6)	(6.4)	(4.5)
Speed (fps)		26.0	18.9	9.2	22.2	25.3	**32.1**
(std)		(0.2)	(0.2)	(0.1)	(1.8)	(0.4)	(4.4)

S, Scene; Ave (std), average of all scenes and (standard deviation); MOTA, Multiple Object Tracking Accuracy^58^ (**bold**: best results).

**Inter-camera tracking performance** Table 4 reports the inter-camera association results. DMMA has a higher MCTA than DMMA-nt and DMMA-nc. DMMA-nc performs better than DMMA-nt, but worse than DMMA, thus validating the use of information from the network. The result of DMMA (MCTA 63.9) on scene 3 which has heavy illumination changes can be considered satisfactory, given that no explicit cross-camera calibration or training is performed.

**Table 4:** Performance evaluation for inter-camera association on the proposed dataset.

		DMMA-nt	DMMA-nc	DMMA
MCTA	S1	96.1	95.4	**97.3**
	S2	89.2	82.4	**98.5**
	S3	54.4	57.2	**63.9**
	S4	63.1	75.2	**97.7**
	S5	54.5	64.2	**91.6**
	S6	56.4	59.3	**82.1**
	Ave	68.8	72.3	**88.5**
	(std)	(17.1)	(13.5)	(12.3)
Speed [fps]		23.3	**33.1**	32.4
(std)		(0.3)	(4.4)	(4.2)

S, Scene; Ave (std), average of all scenes and (standard deviation); MCTA, Multi-Camera object Tracking Accuracy^12^ (**bold**: best results).

In terms of speed, DMMA achieves 32 fps, only 1 fps slower than DMMA-nc which does not receive data from the network. Note that DMMA-nc and DMMA have a higher standard deviation due to the variability of the target search performed by FCT. As we performed all the tests with display on for the analysis of the results, we also tested the proposed solution with no display to simulate how the implementation would perform if deployed with no screens (when they are not required or available in a system). In this case, the speed increases by about 24% on average.

**Impact of parameters** Table 5 shows the impact of detection frequent $\delta$ and maintaining frame number *J* on our dataset. As we can observe that too large $\delta$ and *J* lead to degradation of accuracy, which indicates drift caused without the detector’s correction over a long duration. However, smaller $\delta$ results in recalling detector and initializing trackers frequently, which is time-consuming. Consequentially, we set $\delta = 5$ and *J* = 2 to strike a good balance between speed and accuracy. We further perform a sensitivity analysis for $\psi$, $\gamma$, $\alpha _f$ and $\alpha _n$, and, on average, results remain substantially unchanged in our experiments with a 10% variation.

**Table 5:** MCTA of different $\delta$ and *J* on the proposed dataset (**bold**: best results).

$$\delta$$					
*J*	3	4	5	6	7
1	91.0	91.1	90.4	90.5	89.7
2	**91.9**	91.8	91.6	90.2	89.3
3	91.1	90.8	89.8	90.3	89.6

**Performance on MOT16** We compare DMMA-nc with state-of-the-art MOT trackers including one-shot (FairMOT) and two-step (DeepSort^29^ and MFI_tst^35^) MOT trackers. Following FairMOT^34^, we pre-train the detector on the CrowdHuman dataset^60^. Table 6 shows the performance results. Due to the robustness of proposed target representation, we have the lowest IDs within comparative trackers. This demonstrates that we obtain consistent trajectories of objects. Also, DMMA-nc has the second highest MOTA score and IDF1. This can be attributed to the proposed target management maintaining object association in spite of occlusions and entrance/exiting of camera FoVs. Although FairMOT out-performances DMMA-nc in MOT metrics, the main contribution of DMMA is to devise a data association among mobile camera network without cross-camera calibration.

**Table 6:** Comparison of MOT trackers on MOT16 dataset ($\downarrow$ = the lower the better; and $\uparrow$ = the higher the better; **bold**: best results).

Trackers	MOTA$\uparrow$	IDF1$\uparrow$	MT$\uparrow$	ML$\downarrow$	IDs$\downarrow$
DeepSort^29^	61.4	62.2	32.8	18.2	781
MFI_tst^35^	59.8	58.7	24.1	30.8	617
FairMOT^34^	**74.9**	**72.8**	**44.7**	**15.9**	1074
DMMA-nc	63.2	64.7	36.5	16.4	**523**

**Figure 6:**
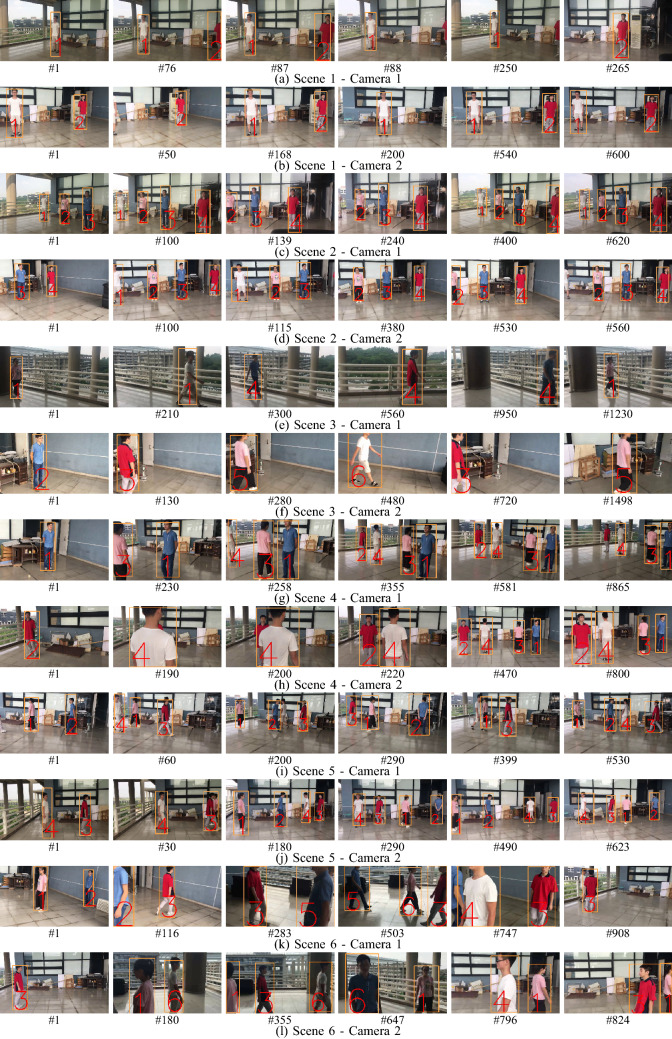
DMMA results on the proposed dataset. Different scenes show different frame numbers to better demonstrate the challenging scenarios.

**Qualitative results** Finally, qualitative results are shown in Fig. 6. In Fig. 6e, f, we can appreciate the heavy illumination change in Scene 3 that leads to a wrong label assignment in Camera 1 while tracking performs well in Camera 2. In Fig. 6h, although Target 2 is completely occluded by Target 4, the method can properly assign the correct label. Similarly, in Fig. 6k the correct labels are assigned even when the targets are not entirely visible. However labels 5 and labels 6 are wrongly assigned due to the very dark conditions created in the scene.

## Conclusion

We presented a target-management module for multi-camera multi-target tracking for a moving-camera network that runs in real-time reaching 32 fps on HD videos. The tracker, DMMA, allows cameras to join or leave without affecting the network’s performance along with targets that are re-identified when re-entering the camera’s FOVs. The tracker can also deal with heavy occlusions and targets at different scales. Experiments were performed on a new mobile-camera dataset and public MOT dataset. Experiment results demonstrate the proposed approach performs well in terms of accuracy, effectiveness and speed.

As future work, we will extend the validation to other camera networks with a variable number of cameras and with a real communication channel.

### Informed consent

For online open-access publication of the images has been obtained from all the participants.

## Data Availability

The datasets used and analysed during the current study available from the corresponding author on reasonable request.

## References

[1] Esterle, L. & Lewis, P. Online multi-object k-coverage with mobile smart cameras. In *International Conference on Distributed Smart Cameras* Stanford, USA (2017).

[2] Wang X (2013). Intelligent multi-camera video surveillance: A review. Pattern Recogn. Lett..

[3] Altan, A. & Hacioğlu, R. The controller of the camera used in target tracking for unmanned vehicle with model predictive controller. In *2014 22nd Signal Processing and Communications Applications Conference (SIU)* 1686–1689 (IEEE, 2014).

[4] SanMiguel JC, Cavallaro A (2017). Networked computer vision: The importance of a holistic simulator. Computer.

[5] Altan A, Hacıoğlu R (2020). Model predictive control of three-axis gimbal system mounted on UAV for real-time target tracking under external disturbances. Mech. Syst. Signal Process..

[6] Wang X, Wang S (2007). Collaborative signal processing for target tracking in distributed wireless sensor networks. J. Parallel Distrib. Comput..

[7] Kuhn HW, Yaw B (1955). The hungarian method for the assignment problem. Nav. Res. Logist. Q..

[8] Saeed F (2021). A robust approach for industrial small-object detection using an improved faster regional convolutional neural network. Sci. Rep..

[9] Yang S (2019). Visual object tracking robust to illumination variation based on hyperline clustering. Information.

[10] He, S., Luo, H., Wang, P., Wang, F., Li, H. & Jiang, W. Transreid: Transformer-based object re-identification. In *2021 IEEE/CVF International Conference on Computer Vision (ICCV)* 14993–15002 10.1109/ICCV48922.2021.01474 (2021).

[11] Taj M, Cavallaro A (2011). Distributed and decentralized multicamera tracking. IEEE Sig. Proc. Mag..

[12] Chen W, Cao L, Chen X, Huang K (2017). An equalized global graph model-based approach for multi-camera object tracking. IEEE Trans. Circuits Syst. Video Technol..

[13] Anjum, N. & Cavallaro, A. Trajectory association and fusion across partially overlapping cameras. In *IEEE International Conference on Advanced Video and Signal Based Surveillance*, Genova, Italy (2009).

[14] Chen Y, Zhao Q, An Z, Lv P, Zhao L (2016). Distributed multi-target tracking based on the K-MTSCF algorithm in camera networks. IEEE Sens. J..

[15] Liu G, Tian G, Li J, Zhu X, Wang Z (2018). Human action recognition using a distributed rgb-depth camera network. IEEE Sens. J..

[16] Qu, W., Schonfeld, D. & Mohamed, M. Decentralized multiple camera multiple object tracking. In *IEEE International Conference on Multimedia and Expo*, Toronto, Canada (2006).

[17] Schwager M, Julian BJ, Angermann M, Rus D (2011). Eyes in the sky: Decentralized control for the deployment of robotic camera networks. Proc. IEEE.

[18] Cai, Y. & Medioni, G. Exploring context information for inter-camera multiple target tracking. In *IEEE Winter Conference on Applications of Computer Vision*, Steamboat Springs, USA 761–768 (2014).

[19] Hofmann, M., Wolf, D. & Rigoll, G. Hypergraphs for joint multi-view reconstruction and multi-object tracking. In *IEEE Conference on Computer Vision and Pattern Recognition*, Portland, USA (2013).

[20] Li Y, Wang S, Tian Q, Ding X (2015). Feature representation for statistical-learning-based object detection: A review. Pattern Recogn..

[21] Sezer, A. & Altan, A. Detection of solder paste defects with an optimization-based deep learning model using image processing techniques. Soldering & Surface Mount Technology (2021).

[22] Liu, W., Anguelov, D., Erhan, D., Szegedy, C., Reed, S., Fu, C. Y. & Berg, A. C. SSD: Single shot multibox detector. In *European Conference on Computer Vision* 8–16 Oct 2016, Amsterdam, The Netherlands (2016).

[23] Redmon, J. & Farhadi, A. Yolov3: An incremental improvement. CoRR arXiv:1804.02767 (2018).

[24] Sandler, M., Howard, A., Zhu, M., Zhmoginov, A. & Chen, L. C. Mobilenetv2: Inverted residuals and linear bottlenecks. In *2018 IEEE/CVF Conference on Computer Vision and Pattern Recognition* 4510–4520 10.1109/CVPR.2018.00474 (2018).

[25] Tan, M., Pang, R. & Le, Q. V. Efficientdet: Scalable and efficient object detection. In *Proceedings of the IEEE/CVF Conference on Computer Vision and Pattern Recognition (CVPR)* (2020).

[26] Choi W, Pantofaru C, Savarese S (2013). A general framework for tracking multiple people from a moving camera. IEEE Trans. Pattern Anal. Mach. Intell..

[27] Xiang, Y., Alahi, A. & Savarese, S. Learning to track: Online multi-object tracking by decision making. In *IEEE International Conference on Computer Vision* 11–18 Dec 2015, Las Condes, Chile (2015).

[28] Bewley, A., Ge, Z., Ott, L., Ramos, F. & Upcroft, B. Simple online and realtime tracking. In *IEEE International Conference on Image Proceedings*, Phoenix, USA (2016).

[29] Wojke, N., Bewley, A. & Paulus, D. Simple online and realtime tracking with a deep association metric. In *IEEE International Conference on Image Proceedings*, Beijing, China 10.1109/ICIP.2017.8296962 (2017).

[30] He, J., Huang, Z., Wang, N. & Zhang, Z. Learnable graph matching: Incorporating graph partitioning with deep feature learning for multiple object tracking. In *Proceedings of the IEEE/CVF Conference on Computer Vision and Pattern Recognition (CVPR)* 5299–5309 (2021).

[31] He Q, Sun X, Yan Z, Li B, Fu K (2022). Multi-object tracking in satellite videos with graph-based multitask modeling. IEEE Trans. Geosci. Remote Sens..

[32] Stadler, D. & Beyerer, J. Improving multiple pedestrian tracking by track management and occlusion handling. In *Proceedings of the IEEE/CVF Conference on Computer Vision and Pattern Recognition (CVPR)* 10958–10967 (2021).

[33] Liu Q (2022). Online multi-object tracking with unsupervised re-identification learning and occlusion estimation. Neurocomputing.

[34] Zhang Y, Wang C, Wang X, Zeng W, Liu W (2021). Fairmot: On the fairness of detection and re-identification in multiple object tracking. Int. J. Comput. Vision.

[35] Yang J, Ge H, Yang J, Tong Y, Su S (2022). Online multi-object tracking using multi-function integration and tracking simulation training. Appl. Intell..

[36] Kviatkovsky I, Adam A, Rivlin E (2013). Color invariants for person reidentification. IEEE Trans. Pattern Anal. Mach. Intell..

[37] Ma L, Tan T, Wang Y, Zhang D (2003). Personal identification based on iris texture analysis. IEEE Trans. Pattern Anal. Mach. Intell..

[38] Wang, X., Doretto, G., Sebastian, T., Rittscher, J. & Tu, P. Shape and appearance context modeling. In *IEEE International Conference on Computer Vision*, 14–20 Oct 2007, Rio de Janeiro, Brazil (2007).

[39] Ahmed, E., Jones, M. & Marks, T. K. An improved deep learning architecture for person re-identification. In *IEEE Conference on Computer Vision and Pattern Recognition*, Boston, USA, 7–12 June 2015 (2015).

[40] Wu, Y., Li, W., Minoh, M. & Mukunoki, M. Can feature-based inductive transfer learning help person re-identification? In *IEEE International Conference on Image Proceedings*, Melbourne, Australia 2812–2816 (2013).

[41] Peng, P., Xiang, T., Wang, Y., Pontil, M., Gong, S., Huang, T. & Tian, Y. Unsupervised cross-dataset transfer learning for person re-identification. In *IEEE Conference on Computer Vision and Pattern Recognition* 26 Jun–1 Jun 2016, Las Vegas, USA (2016).

[42] Cheng D (2018). Cross-scenario transfer metric learning for person re-identification. Pattern Recogn. Lett..

[43] Wang, J., Zhu, X., Gong, S. & Li, W. Transferable joint attribute-identity deep learning for unsupervised person re-identification. In *IEEE Conference on Computer Vision and Pattern Recognition*, Salt Lake City, USA (2018).

[44] Henriques, J. F., Rui, C., Martins, P. & Batista, J. Exploiting the circulant structure of tracking-by-detection with kernels. In *European Conference on Computer Vision*, 7–13 Oct 2012, Firenze, Italy (2012).

[45] Bertinetto, L., Valmadre, J., Golodetz, S., Miksik, O. & Torr, P. H. S. Staple: Complementary learners for real-time tracking. In *IEEE Conference on Computer Vision and Pattern Recognition*, Las Vegas, USA 1401–1409 (2016).

[46] Bof N, Carli R, Cenedese A, Schenato L (2017). Asynchronous distributed camera network patrolling under unreliable communication. IEEE Trans. Autom. Control.

[47] Weijer JVD, Schmid C, Verbeek J, Larlus D (2009). Learning color names for real-world applications. IEEE Trans. Image Proc..

[48] Danelljan, M., Khan, F. S., Felsberg, M. & Weijer, J. V. D. Adaptive color attributes for real-time visual tracking. In *IEEE Conference on Computer Vision and Pattern Recognition* 24–27 Jun 2014, Columbus, USA (2014).

[49] Kim, M., Jung, J., Kim, H. & Paik, J. Person re-identification using color name descriptor-based sparse representation. In *IEEE Annual Computing and Communication Workshop and Conference* Las Vegas, USA (2017).

[50] Martinel, N. & Micheloni, C. Sparse matching of random patches for person re-identification. In *International Conference on Distributed Smart Cameras* 4–7 Nov 2014, Venezia, Italy (2014).

[51] Ye M, Shen J, Lin G, Xiang T, Shao L, Hoi SCH (2021). Deep learning for person re-identification: A survey and outlook. IEEE Trans. Pattern Anal. Mach. Intell..

[52] Ferryman, J. & Shahrokni, A. Pets2009: Dataset and challenge. In *IEEE International Workshop on PERFORMANCE Evaluation of Tracking and Surveillance*, 7–9 Dec 2009, Snowbird, USA 1–6 (2009).

[53] Ristani, E., Solera, F., Zou, R., Cucchiara, R. & Tomasi, C. Performance measures and a data set for multi-target, multi-camera tracking. In *European Conference on Computer Vision*, 8–16 Oct 2016, Amsterdam, The Netherlands 17–35 (2016).

[54] Dollar P, Wojek C, Schiele B, Perona P (2012). Pedestrian detection: An evaluation of the state of the art. IEEE Trans. Pattern Anal. Mach. Intell..

[55] Zhang K, Zhang L, Yang MH (2014). Fast compressive tracking. IEEE Trans. Pattern Anal. Mach. Intell..

[56] Lienhart, R. & Maydt, J. An extended set of haar-like features for rapid object detection. In *International Conference on Image Proceedings* Rochester, USA (2002).

[57] Li, W., Zhao, R., Xiao, T. & Wang, X. Deepreid: Deep filter pairing neural network for person re-identification. In *2014 IEEE Conference on Computer Vision and Pattern Recognition (CVPR)* 152–159 (IEEE Computer Society, Los Alamitos, CA) 10.1109/CVPR.2014.27 (2014).

[58] Bernardin K, Stiefelhagen R (2008). Evaluating multiple object tracking performance: The CLEAR MOT metrics. Eurasip J. Image Video Proc..

[59] Zoph, B., Vasudevan, V., Shlens, J. & Le, Q. V. Learning transferable architectures for scalable image recognition. In *2018 IEEE/CVF Conference on Computer Vision and Pattern Recognition* 8697–8710 10.1109/CVPR.2018.00907 (2018).

[60] Shao, S., Zhao, Z., Li, B., Xiao, T., Yu, G., Zhang, X. & Sun, J. Crowdhuman: A benchmark for detecting human in a crowd arXiv:1805.00123 (2018).

